# Passenger physiology in self-driving vehicles during unexpected events

**DOI:** 10.1038/s41598-024-81960-4

**Published:** 2025-03-06

**Authors:** Zsolt Palatinus, Miklós Lukovics, Márta Volosin, Zsolt Dudás, Szabolcs Prónay, Zoltán Majó-Petri, Henrietta Lengyel, Zsolt Szalay

**Affiliations:** 1 Department of Neuroscience and Cognition, Institute of Psychology, University of Szeged, Egyetem utca 2, Szeged, 6722 Hungary; 2 Research Centre for Natural Sciences, Institute of Cognitive Neuroscience and Psychology, Magyar Tudósok körútja 2, Budapest, 1117 Hungary; 3 Department of Economic Development, Faculty of Economics and Business Administration, University of Szeged, Szeged, Hungary; 4 Department of Business Studies, Faculty of Economics and Business Administration, University of Szeged, Szeged, Hungary; 5 Department of Automotive Technologies, Budapest University of Technology and Economics, Budapest, Hungary

**Keywords:** Self-driving, Technology acceptance, EEG, Eye-tracking, Wearable sensors, Multiscale analysis, Neuroscience, Physiology, Psychology, Engineering

## Abstract

While using fully autonomous vehicles is expected to radically change the way we live our daily lives, it is not yet available in most parts of the world, so we only have sporadic results on passenger reactions. Furthermore, we have very limited insights into how passengers react to an unexpected event during the ride. Previous physiological research has shown that passengers have lower levels of anxiety in the event of a human-driven condition compared to a self-driving condition. The aim of our current study was to investigate these differences in unexpected road events in real-life passenger experiences. All subjects were driven through a closed test track in human-driven and then self-driving mode. During the journey, unforeseen obstacles were encountered on the path (deer and human-shaped dummies appeared). Using physiological measurements (EEG, eye movements, head movements and blinking frequencies) our results suggest that passengers had moderate affective preferences for human-driven conditions. Furthermore, multifractal spectra of eye movements and head movements were wider and blinking frequencies were decreased during unexpected events. Our findings further establish real-world physiological measurements as a source of information in researching the acceptance and usage of self-driving technologies.

## Introduction

It has been argued that the most challenging roadblocks towards widespread acceptance of self-driving vehicles could be more social than technical in nature^1–4^. Perception of usefulness^5,6^, trust^7,8^, safety^9,10^ have all been identified as important factors that influence acceptance. Investigating the perceptual factors of technology acceptance, however, has its own challenges. Perception is rather an umbrella term, as it may refer to the opinion of the user, feelings, ideas, attitudes colored by personality traits, previous experiences, desires etc^11,12^. These factors to this day are commonly measured using questionnaires or interviews^13,14^. However, from a more biological or ecological perspective, perception refers to awareness of factors of the environment that have significance with respect to our goals, actions, and well-being. Most importantly, perceptual processes involve the visual and auditory system the central nervous system (CNS) and they are closely coupled to cardiovascular, respiratory, and motor systems. This strongly suggests that an effective research method investigating factors of perception and acceptance of AV technologies must include physiological measurements.

In a previous pilot study, electroencephalography (EEG) and eye-movement data were collected in a 3-minute-long real-world driving situation^15^. Participants in the front passenger seat experienced both self-driving and human driving modes. In the analyses, both measurement types signaled a difference between traditional and self-driving: participants’ physiological signals, such as frontal alpha asymmetry and multifractal spectrum, suggested a higher preference for human drivers in contrast to the autonomous driving mode. However, even for a road trip this short, the experience is not homogeneous. During a road trip, passengers experienced regular or expected events such as acceleration, deceleration, and changing lanes following the curvature of the pavement. Irregular or less expected events may also occur when sudden deceleration or acceleration or quick path alterations are necessary to avoid collisions. A passenger, being affected by the movement of the vehicle, may form conscious or unconscious expectations about the dynamics of these events. The extent to which these expectations are being met could be a factor in the perception of safety and usefulness of any given mode of transportation^16,17^.

Our aim in the current investigation was to test human-driven and self-driving scenarios with the addition of unexpected events to passenger experience. Our main question was how a combination of complex physiological measures could be used to identify differences in reactions to unexpected situations when comparing self-driving with human-driven scenarios. For this experiment we utilized a car that had a self-driving system more closely based on human driving compared to the one we used in our pilot^15^. Even though experimental assessment of physiological and psychological responses to different types of self-driving technologies is outside of the scope of the current study, our analyses may serve as starting points towards such investigations. For adding unexpected events, life size dummies made of plastic and other relatively collision-safe materials were placed on the side of the road, prompting quick evasion maneuvers in both human-driven and self-driving conditions. We expected these events to either override the effect of driving conditions or interact with them in ways that allow us to draw conclusions about the possible moderating effect of the unexpected events on the physiology of the passengers. If measured differences between self-driving and human driving conditions get moderated or eliminated by unexpected events, it may signal an important message to developers and users alike: firsthand experiences of safety and the ability to handle critical situations on the road may be an essential factor in technology acceptance. In the following, we enumerate the main characteristics of the measurements we utilized during the data collection as well as the most important results from the literature and articulate our hypotheses.

## EEG

In the past decade, there has been a rapid growth in the availability of portable sensors, making it possible to design ecologically valid field studies to measure physiological signals such as eye movements or the electric activity of the brain^18^. EEG signals consist of different frequency ranges, and their ratio allows one to draw conclusions about changes in one’s mental and emotional state over the recording^19^.

Numerous studies demonstrated that higher frequency oscillations reflect more alerted and aroused states when the power ratio of higher (beta: 13–30 Hz, gamma: 30–45 Hz) and lower frequencies (alpha: 8–12 Hz, theta: 4–8 Hz) are compared^20,21^. In contrast, lower frequencies dominate when the participant is relaxed. For example, concentrated attention was correlated with higher relative gamma activity^21^, and enhanced stress level was reflected by increased beta/alpha^21,22^, decreased alpha/beta and theta/beta^23^ or increased relative gamma ratio^24^.

In addition to arousal, a further well-studied area of EEG band signatures is to follow-up emotional and motivational states. Based on the hemispherical (left-minus-right) differences in the alpha power at frontal electrodes (frontal alpha asymmetry):^25,26^, higher values indicate more positive or approaching attitudes, while lower values indicate more negative or withdrawal attitudes^26–28^. Frontal alpha asymmetry was found not only to be a good primer of depression^26^ but also an indicative tool in applied sciences^29^.

Both arousal and frontal alpha asymmetry were used in studies investigating drivers’ or passengers’ reactions to unexpected road events, mostly in simulated tasks. For example, when the driver detected a hazard cue, the power of the alpha band immediately decreased, and the beta power increased approximately 300 ms after the cue appeared^30^. Similarly, when a ship driver could not avoid a collision, higher frequencies dominated in comparison to successful collision avoidance^31^. Explicitly focusing on self-driving vehicles, in a case study^32^, participants were presented with positive (smooth highway driving) and negative (erratic driving and violating common rules of the road) driving situations. The beta-to-alpha power ratio increased in the negative scenario, suggesting elevated stress levels when being exposed to hazardous driving. Similarly, when participants were facing take-over situations in an autonomous vehicle simulator, they subjectively rated the multi-modal warning signals as the most effective on a Likert-scale. Moreover, these warning signals were predominantly accompanied by the presence of higher frequencies in EEG^21^.

Regarding emotional valence^33^, when malfunctions appeared during a simulated drive in an autonomous vehicle, frontal alpha power reduction was present in the right but not in the left hemisphere during the fully automated condition compared to when participants were able to control the vehicle. The authors interpreted this effect as an enhanced motivation of the driver towards controlling the vehicle, which was in line with participants’ explicitly verbalized preferences^33^. In a further study utilizing narrow vehicles, higher arousal and higher frontal alpha asymmetry values were measured when the vehicle was able to tilt in curved sections of the road, which was in line with subjective evaluations reflecting user satisfaction^34^. Comparable results were found when instead of a traffic situation, wheelchair users were sitting in an autonomous wheelchair driving across a narrow/constrained or a wide/open path. Narrow paths resulted in frontal alpha asymmetry pattern related to avoidance during these hazardous situations^35^.

## Eye movements and head movements

Recently, more focused analyses have targeted eye and head movements in the context of developing driver assistance systems and self-driving navigation technologies. Drivers and passengers are surrounded by a highly dynamic, rapidly changing environment in which the visual information that is relevant for navigation must be sampled in specific ways, often including a wide variety of eye and head movements^36^. It has been pointed out that despite being linked to distinct neuromuscular systems, eye and head movements are highly correlated^37^ and are a source of an extensive amount of information about the driver or the passenger. In a more general context, fluctuations in movements of the eyes, head or hands could be harnessed as an information “substrate” spreading across the perceptual/motor system linked to coordination and cognition^38^. In this line of research, multifractal analysis of movement data (more precisely, high rate sampling of posture, head, hands or eye displacement) has been repeatedly shown to signal cognitive transitions and processes, including problem-solving^39^, magnitude perception^40^, perceptual intent^41^, visual recognition^42^, comprehension^43^, and memory^44^. In our previous study^15^, these measures also indicated a connection to anxiety and heightened awareness among passengers traveling in self-driving modes. Focusing on fluctuations and displacement data also facilitates non-invasive, real-world research methods that allow for experimentation in close to real-life circumstances. Besides complex movement patterns, relatively simple changes in spontaneous blink frequency (SBF) have been reported to show correlation with anxiety and novel stimulation^45,46^.

## Hypotheses

The goal of the present study was to investigate the fluctuation changes in physiological signals of a passenger when unexpected road events appear and the potential differences between situations when a human person drives the car (Human condition) versus when the vehicle requires no manual input from a human driver to complete the track (Self-driving condition).

For the EEG measurement, we hypothesized lower frontal alpha asymmetry values in the self-driving condition. We also hypothesized higher arousal and lower frontal alpha asymmetry values to unexpected road events. For the eye-, and headtracking data, we expected to see the effect of novelty and anxiety manifest in a narrower multifractal spectrum for the self-driving conditions countered by the contribution of unexpected events where a heightened need for visual information may result in broader spectra. For the same reason, blinking frequencies were expected to show lower values for the novel and unexpected stimulation.

## Methods

### Participants and procedure

41 healthy adult volunteers (mean age: 39.175 years, SD = 11.200 years, from 21 to 65 years, 18 females, 3 left-handed) participated in the present study. All of them reported normal or corrected-to-normal vision and hearing and no psychiatric or neurological problems. Participants were recruited via social media, and they received no monetary compensation. They gave written informed consent prior to the study. The experiment was conducted in accordance with the Declaration of Helsinki and the protocol was approved by the United Ethical Review Committee for Research in Psychology (EPKEB), Hungary under ref. number SZTE-PI 2022-71(2021-70, 2020-89). The size of the sample used in this study was based on research resources and the number of participants in similar experiments^15,33,47^.

Before and after the experiment, participants were required to fill personality and demographic questionnaires via an online form. As the main scope of the present study is to address differences in the physiological signals during the ride, the data from the surveys will be presented elsewhere.

The experiment took place at ZalaZONE vehicle testing environment in Zalaegerszeg, Zala county, Hungary, on the 30th and 31st of May 2022. ZalaZONE^48^ was proved to be the most suitable test environment for testing self-driving vehicles in Hungary for the two-day test series. The ZalaZONE proving ground features a high-speed handling course, with dimensions of 2000 m in length and 12 m in width, and a soft gravel/asphalt run-off area supported by an 80 cm basalt foundation. This module is ideal for testing and developing vehicles under different driving conditions due to its varied topography and ability to simulate real-world driving scenarios. It is particularly suitable for chassis development, steering, damping, braking, and acceleration maneuvers required in the vehicle and tire development industry, as well as advanced driver assistance systems and automated driving test scenarios. The course can also be used for inter-module testing, enabling various traffic situations to be simulated. The vehicle was KIA Negra test car, modified by ZalaZONE to be able to navigate the entire track without any help or manual input from the driver. In short, the modified vehicle was operated through its electronic actuators, which executed the pre-recorded movements of a human driver.

**Fig. 1:**
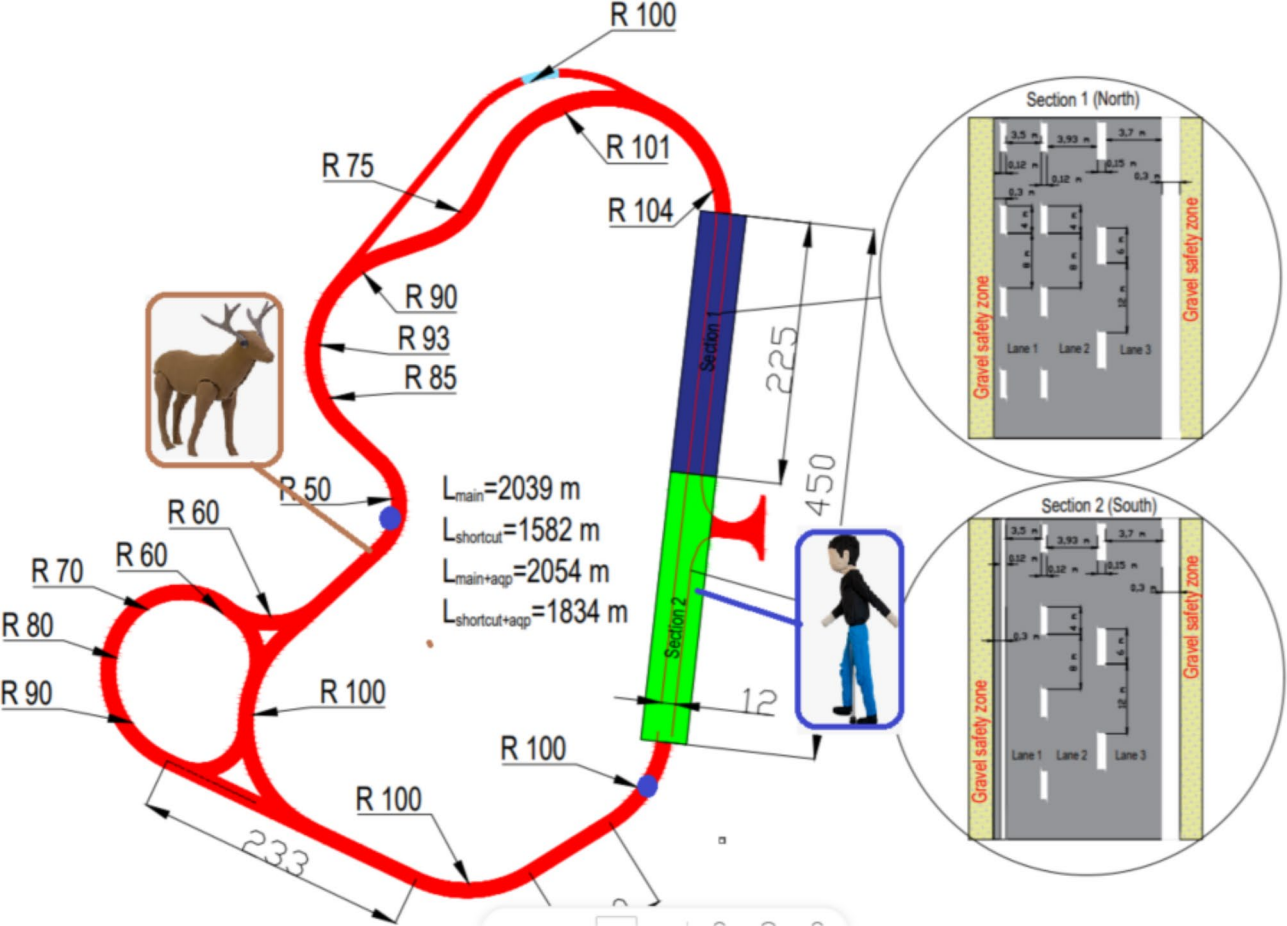
Overhead view of the route utilized in the study. The blue dots indicate the positions of the two dummies.

To establish unexpected driving situations, two dummies were placed on the road that the vehicle had to avoid. The two blue dots in Fig. 1. indicate the position of the two dummies. These dummies were devices used during official ISO 19206-2, Euro NCAP, CNCAP, JNCAP tests, suitable for testing under rough conditions. The deer weighed 7.5 kg and measured 1490 × 1210 × 270 mm. The child dummy weighed approx. 4 kg and was 1154 mm tall. The unexpected event or “encounter” happened as the vehicle approached the dummies and made a quick path modification to avoid collision. The maneuver was safe for a participant wearing a seatbelt. It was an imitation of an event that did not prompt emergency actions but required path correction.

When participants arrived at the test field, they were debriefed about the goal and the schedule of the experiment. They gave informed consent for the experiment, and completed a short questionnaire, then EEG, eye-tracking glasses and smart watch were mounted on them.

After that, the participant and two experimenters holding separate portable laptops for EEG and eye tracking signals were seated in the vehicle. The driver was seated in the driver’s seat (front left) and the participant was seated in the passenger’s seat (front right). The two experimenters were seated in the back seat, operating the recording equipment. Participants were instructed to behave like a passenger in general while sitting relaxed and minimizing head and body movement. The route was taken twice: first, a professional driver was driving (Human condition), and in the second round, the self-driving mode was switched on and the driver released the steering wheel (Self-driving condition) or vice versa.

In the self-driving condition, the self-driving mode worked under the supervision of the operator. The vehicle stayed in self-driving mode as long as the operator was holding the Deadman switch. When the operator deemed a situation risky, he could take control of the vehicle by releasing this switch. In addition, there was a red Emergency button in the vehicle, which, when pressed, also deactivated self-driving. The vehicle travelled at a maximum speed of 60 km/h.

The order of human and self-driving conditions was counterbalanced between participants: 21 participants started with the human, and 20 participants started with the self-driving condition. Both types of blocks lasted about 3 min and were recorded separately. Before and after the two runs, participants completed a set of questionnaires. The whole session at the testing environment took about an hour per participant.

### Measures and data analysis

#### EEG

Continuous EEG data was recorded with a portable OpenBCI 4-channel Ganglion board utilizing Lab Streaming Layer (LSL) from OpenBCI GUI at a 200 Hz sampling rate. Four gold cup electrodes were attached to participants’ scalp with conductive paste (Ten20) in accordance with the 10–20 system^49^ to F3, F4, FPz and Oz. Two additional electrodes were attached to the left and right mastoids serving as reference and ground electrodes, respectively. Impedances were kept below 30 kΩ.

##### Preprocessing data

On the continuous EEG data, independent component analysis (ICA) was applied to detect and correct artefacts such as eye movements, muscular or heart signals by the FastICA algorithm of scikit-learn Python module (version 1.1.0;^50^). The artefact-corrected EEG was filtered offline; a bandpass filter (7–48 Hz, 9th order Butterworth) was applied as this range characterized the frequencies of our interest. After filtering, continuous data was segmented into 2-second long epochs with 1-second overlapping parts. Epochs with a signal range exceeding ± 100 µV (typically due to movement or blink artefacts) were excluded from further analysis.

Power spectral density (PSD) values were calculated for alpha (8–12 Hz), beta (13–30 Hz) and gamma (30–45 Hz) frequency bands by applying the Welch method. Two indices were calculated for each epoch: first, affectivity as indexed by frontal alpha asymmetry was computed as the difference between log10 transformed values of F4 and F3^25,26^. Second, arousal was defined as the ratio of PSD in the beta and gamma range to the alpha range at the averaged F3 and F4 electrodes. Higher values represent more positive emotional valence^24–26^ and higher arousal^20,21^. Outlier epochs that deviated from the participant’s individual mean by at least 3 standard deviations (SD) were removed before averaging data for both affectivity and arousal. Data was averaged into four segments based on the position of the dummies. The deer dummy was placed at about 50% of the route and the human pedestrian was placed at about 90% of the route. These spots were used as anchors for segmenting the data into different road events. The first segment started at the beginning of the route and lasted until the deer dummy minus 5 s (1000 time points). The second segment started 5 s before the deer dummy and lasted until 60% of the block. The third segment started at 60% of the block and lasted until the human pedestrian dummy minus 5 s. The fourth segment started at 5 s before the human pedestrian dummy and lasted until the end of the route. The first and third segments were considered as “smooth” while the second and fourth segments were considered as “unexpected” parts of the ride. The interval of 1000 time points was chosen because the dummies appeared in the visual field approximately at this time.

### Eye tracking

Eye movements were recorded using the Core System manufactured by Pupil Labs (Berlin, Germany) connected to a portable laptop computer (ROG Zephyrus GA410QE). This system has two types of cameras: one is positioned forward to record the field of view of the wearer in HD video at 30 Hz, and a second infrared camera recorded the participant’s eye movements. Pupil Player software developed by the manufacturer (version 3.5, https://pupil-labs.com/products/core/), was used to export the eye movements data for further analysis.

#### Preprocessing data

Real-life eye-tracking data has more noise that those recorded in controlled laboratory environments. Therefore, we improved accuracy using convolution algorithms to detect the pupil area in the raw video data of the eye cameras. The segmentation procedure was set to be as sensitive as possible, i.e., the area that best matched to the pupil was selected as the output of the function. This also caused the system to indicate a match scattered over the entire area of the image, even when no pupil was present^51^.

The center, x y size, and rotation angle of the ellipsis that best fit the segmented areas were determined. Next, the eye coordinates were converted to degrees of rotation by determining the center and extent of the vitreous body (bulbus oculi) and the horizontal rotation of the camera using a line connecting the horizontal eye muscles (m. rectus medialis and m. rectus lateralis). Using these values, the xy pixel coordinates of the eye were converted to YX degrees of rotation^52^. To accept the validity of the measurement, we ignored coordinates that were outside the area of the bulbus oculi and also ignored points between which the speed of the eyeball exceeded 500 degrees/Sect^53^. The omitted points were filled in with 20 ms gaps (cca. 2 frames in 120 Hz sample rate) using linear interpolation in the timeframe. Gaps greater than 20 ms were marked as measurement errors. In the case where the time intervals considered as measurement errors were equal for both eyes, they were labeled as blinks. To suppress the sampling frequency harmonics and ensure proper representation of the signal waveforms, we used a 60 Hz second-order Butterworth low pass filter at the end of the signal processing pipeline.

### Head movement and blinking

To calculate the head movement, the 30 Hz video from the forward pointing camera was used. The images from the camera were compared with the previous images looking for common details, using OpenCV Point Feature Matching From the displacement function. We mapped the points that moved in the same direction and speed, thus determining the total camera movement in x (Horizontal) - y (Vertical) and z (Rotation) directions. We have ignored the effect of pincushion distortion caused by the forward tilt. We also ignored the points which were outside the windscreen area as well as did not follow the movement of most of the points. The resulting displacement coordinates were converted to degrees by knowing the approximate distance of car internals and camera’s angle of view^54^. Although the procedure is not 100% accurate, it is a very strong approximation to determine head displacement.

The recorded pupil and head movement data were segmented according to the events experienced on the handling track. More specifically, three segments were separated: one for normal travel with no unexpected event, one for encountering the deer dummy and one for encountering the human dummy. These segments were equal in length to the collected time series. For each segment, the width of the multifractal spectrum was calculated using the Chhabra and Jensen estimation method^55^ for both eyes, and for head movements in the x and y planes. Blinking frequencies were also summed for each segment.

#### Statistical analysis

For EEG data, we compared overall differences in arousal and affectivity between human and self-driving conditions, and the type of the route events. Separately for arousal and frontal alpha asymmetry values, a two-way repeated measures ANOVA was run: Condition (Human/Self-driving) × Route event (Smooth/Deer/Kid). Similar tests were applied to the multifractal spectrum of eye and head movements and for blinking frequencies. Statistical analyses were conducted in R Statistical language^56^. Generalized eta-square (η^2^_G_) effect sizes^57,58^ are also reported.

## Results

Because of the excessive amount of movement artifacts or data recording issues during the ride (for example, lost connection between the EEG amplifier and the laptop), data of 9 participants had to be excluded from further analysis of EEG data. We excluded data of 7 participants from eye movement, 6 participants of head movement analysis due to low signal confidences.

### EEG affectivity (frontal alpha asymmetry)

**Fig. 2:**
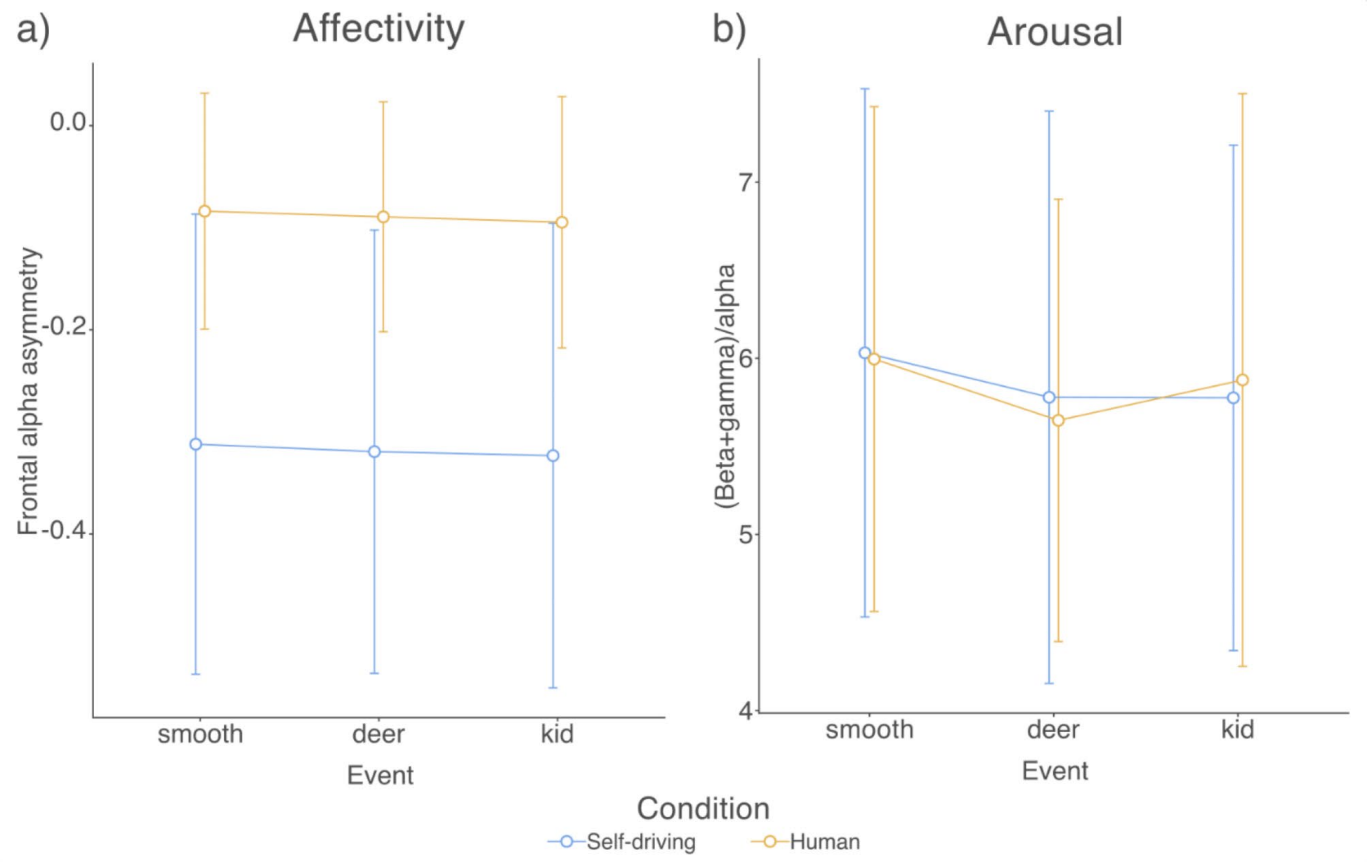
Mean (**a**) frontal alpha asymmetry and (**b**) arousal values plotted by event type and mode of travel. Error bars denote 95% CI.

Regarding frontal alpha asymmetry, a significant main effect of Condition was present (*F*(1, 31) = 5.800, *p* = 0.022, η^2^_G_ = 0.052), suggesting lower values in the Self-driving condition in general. The main effect of Route event (*F*(2, 62) = 0.521, *p* = 0.597, η^2^_G_ < 0.001) and the Condition × Route event interaction (*F*(2, 62) = 0.004, *p* = 0.996, η^2^_G_ < 0.001) interaction were not significant. Frontal alpha asymmetry values are plotted in Fig. 2a.

### EEG arousal

No significant effects were detected in arousal levels. The main effects of the Condition (*F*(1, 31) = 0.004, *p* = 0.950, η^2^_G_ < 0.001) and the Route event (*F*(2, 62) = 1.457, *p* = 0.241, η^2^_G_ < 0.001) were not significant. The Condition × Route event (*F*(2, 62) = 0.121, *p* = 0.886, η^2^_G_ < 0.001) was not significant either. Arousal values are presented in Fig. 2b.

### Eye movements

**Fig. 3:**
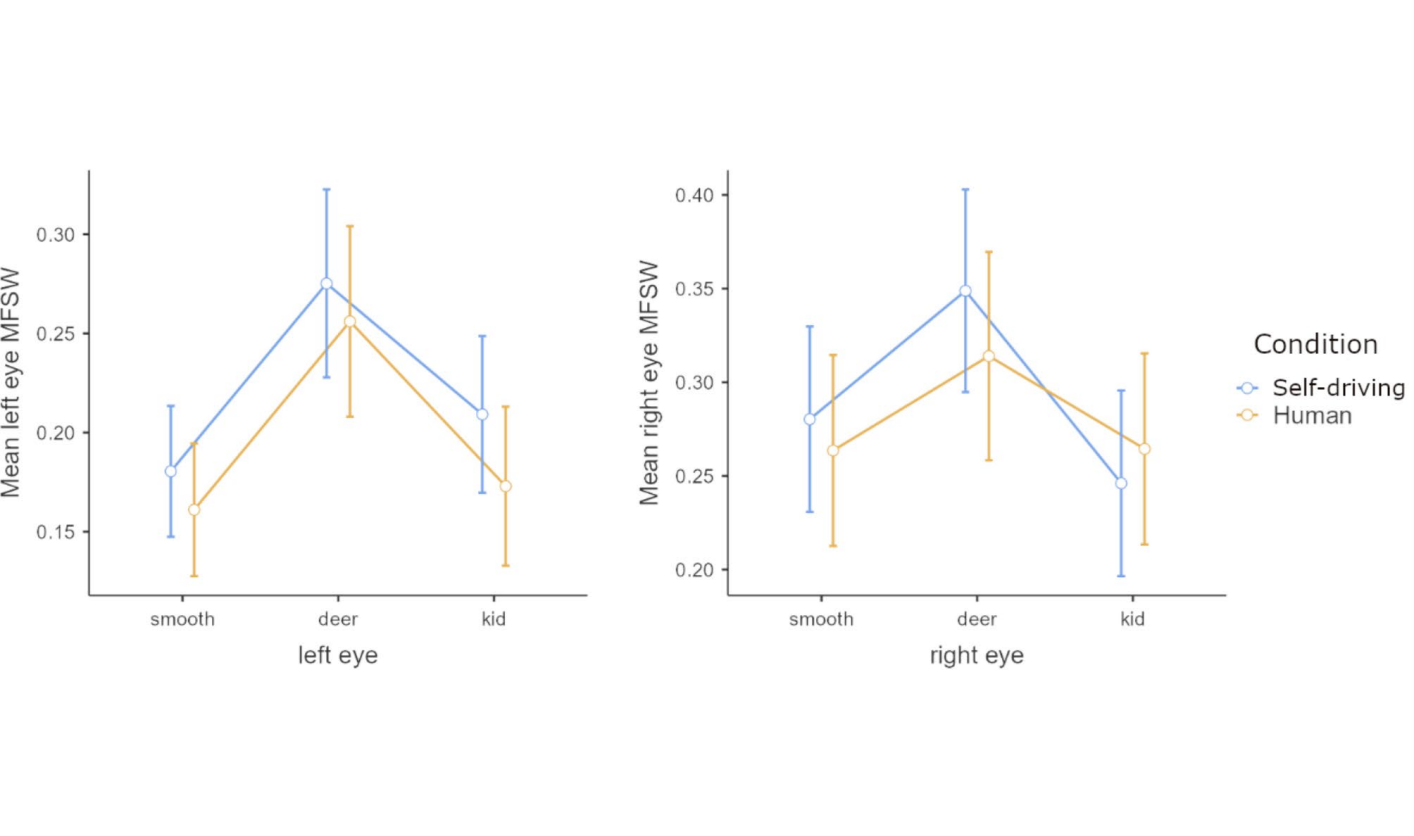
Mean multifractal spectrum width (MFSW) estimates for the left and the right eyes plotted by event type and mode of travel. Error bars denote 95% CI.

For the investigated segments, the main effect of Condition was not significant. There was a significant main effect for Route event (smooth, deer, kid) *F*(2, 68) = 12.416, *p* < 0.001, η^2^_G_ < 0.111 for the left eye and *F*(2, 66) = 5.9, *p* = 0.004, η^2^_G_ = 0.055 for the right eye. For both eyes, post hoc Tukey tests revealed that the encounter with the deer was linked to significantly broader spectra than either of the two other event types for the left eye (smooth vs. deer *t*(33) =-4.38, *p*_*tukey*_ < 0.001; deer vs. kid *t*(33) = 3.84, *p*_*tukey*_ < 0.001). Similar results were found for the right eye as well (smooth vs. deer *t*(33) = -2.606, *p*_*tukey*_ = 0.035; deer vs. kid *t*(33) = 2.98, *p*_*tukey*_ = 0.014). Figure 3 illustrates the overall pattern in multifractal spectrum width in the two Conditions of travel. There was no main effect of the Condition.

### Head movements

**Fig. 4:**
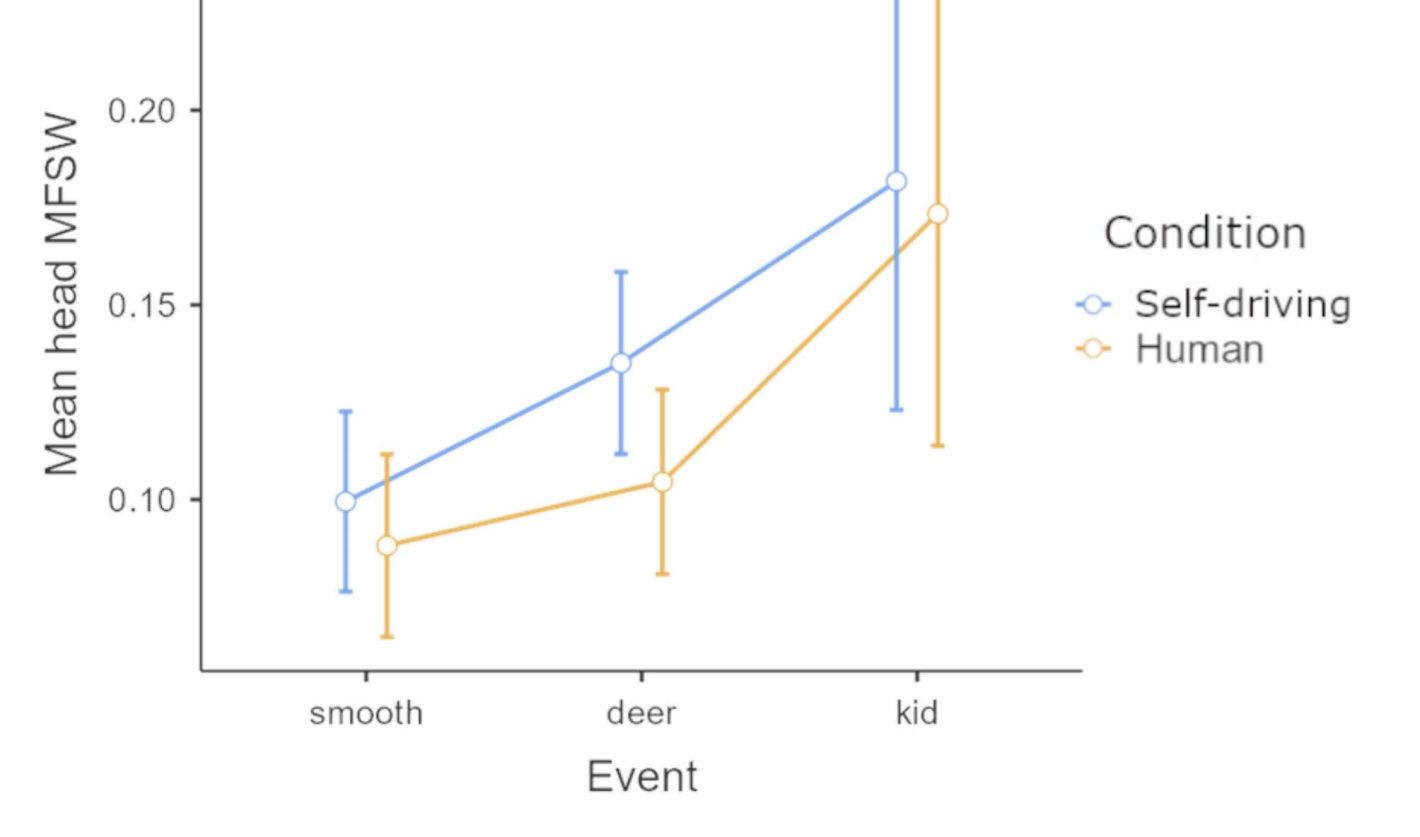
Mean multifractal spectrum width (MFSW) estimates for head movements plotted by event type and mode of travel. Error bars denote 95% CI.

Multifractal spectrum width was significantly different in head movements for event types in both horizontal (x) *F*(2, 68) = 11.057, *p* < 0.001, η^2^_G_ = 0.086 and vertical (y) planes: *F*(2, 68) = 9.09, *p* < 0.001, η^2^_G_ = 0.068. Post hoc Tukey test revealed that both unexpected Route events (deer, kid) were linked to significantly broader spectra than normal traveling in the horizontal plane (smooth vs. deer *t*(34) =-4.071, *p*_*tukey*_ < 0.001, smooth vs. kid *t*(34) =-3.869, *p*_*tukey*_ < 0.001). In the vertical plane Post hoc Tukey test revealed that the difference between no event vs. deer were approaching significance *t*(34) =-2.23, *p*_*tukey*_ = 0.08, smooth vs. kid *t*(34) =-3.92, *p*_*tukey*_ = 0.001 and deer vs. kid *t*(34) =-2.88, *p*_*tukey*_ = 0.018 were both significant. The effect of mode of transportation generated broader spectra in head movement for the Self-driving condition but it was approaching significant in the horizontal plane *F*(1, 34) = 4.04, *p* = 0.052, η^2^_G_ = 0.019. Figure 4 illustrates the overall pattern in the three Conditions. There was no main effect of the Condition. (Fig. 4. here)

### Blinking frequency

**Fig. 5:**
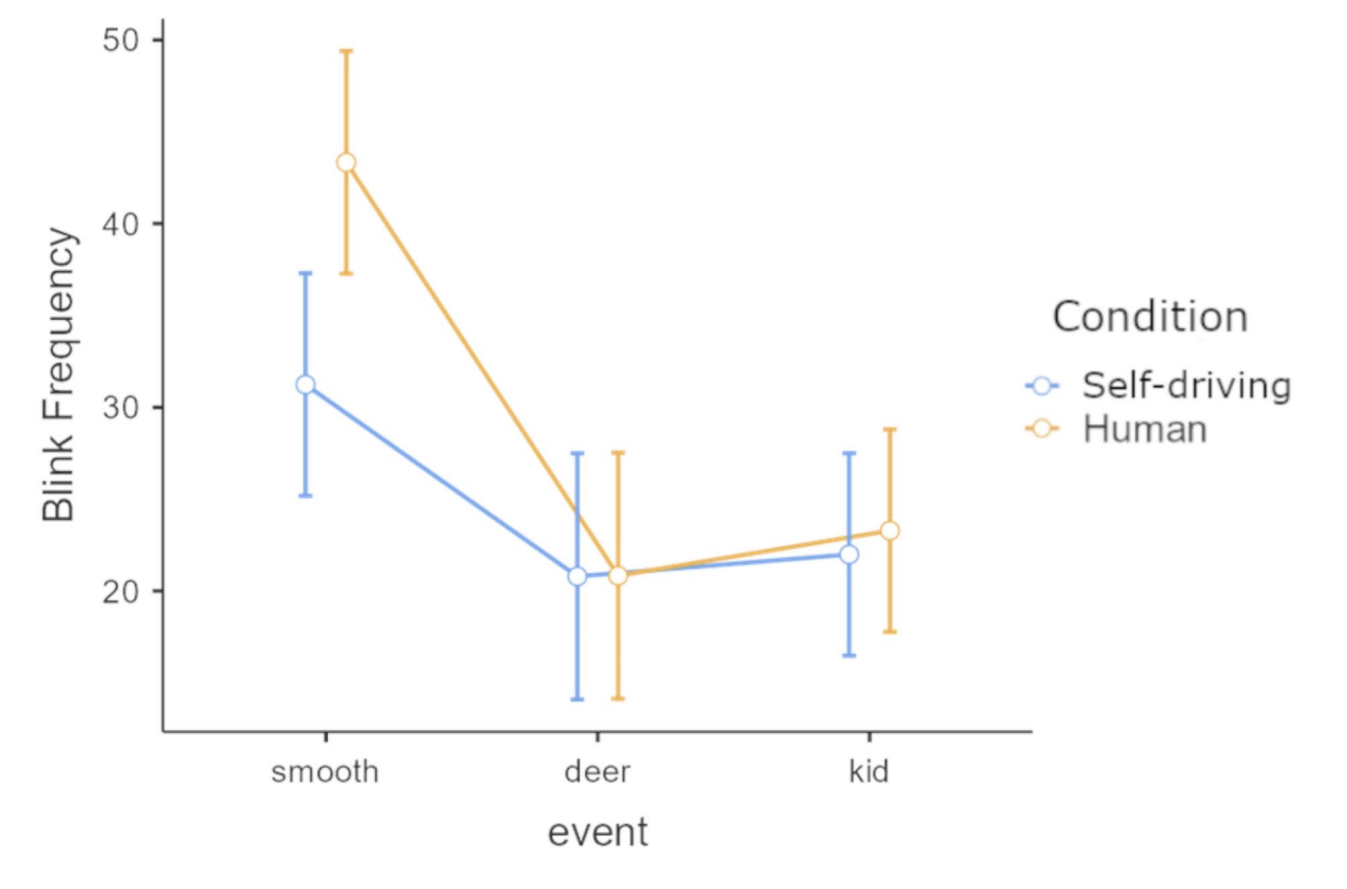
Mean blinking frequencies plotted by event type and mode of travel. Error bars denote 95% CI.

Significantly lower blinking frequencies (main effect of Route event) were recorded during the unexpected events *F*(2, 128) = 33.34, *p* < 0.001, η^2^_G_ = 0.154). The interaction between Route events and travel Conditions was also significant *F*(2, 128) = 4.49, *p* = 0.013, η^2^_G_ = 0.024). This latter interaction was due to the difference between smooth and eventful driving conditions. Figure 5 shows differences of blinking frequency in the two Conditions of travel (Fig. 5 here).

## Discussion

In this study, we aimed to compare reactions to unexpected situations in self-driving versus human-driven scenarios using a combination of complex physiological measures. We recorded and analyzed EEG, eye movement, head movement and blinking frequency in relation to driving conditions and road events. Despite some contrasts, our results were comparable to earlier findings^15^. The expanded design allowed us to see possible interactions between the effect of driving modes and the experienced events.

The most notable result of the EEG analyses showed a significant difference in frontal alpha asymmetry for human and self-driving conditions, suggesting lower affectivity values during the novel experience of the vehicle navigating by itself. This replicates previous findings that participants preferred the situation when the vehicle was driven by a human driver over the self-driving mode^15^. As it was hypothesized earlier, a lack of perceived control over the movement of the vehicle^32,33^ is a possible explanation for the observed disparity here.

Multifractal analysis of both eye movements and head movements indicated a strong effect of the events that demanded quick path corrections from both human and self-driving systems. The sudden need for heightened awareness and more detailed information seeking behavior may be among the reasons for the measured differences along with the need for visual stabilization during the quick evading maneuver. The fact that the spectral structure of the eye movements and the head movements were not identical but indicated a very similar susceptivity to events is a rather promising finding. The previously reported^15^ overall narrower spectrum in eye movements for the self-driving condition was reduced or missing from these results. The absence of significant differences in the multifractal structure of the eye movements may index, among other causes, differences in the navigation mechanics of the utilized self-driving system or the moderating effect of the included events. In the current study, the self-driving vehicle’s steering, accelerating and decelerating behavior were modeled very closely after human driving. A comparison of the velocity profiles of the two driving modes for each event revealed very similar acceleration patterns (average peak difference was 0.02 g). This finding highlights the possibility of using eye-tracking or head-tracking to evaluate self-driving technologies based on how close they “feel” to human driving. Another factor behind the similarity between the modes of transportation could have been the events that prompted evading action in both human and automated driving; diminishing the importance of navigation type in comparison to concerns of safe travel.

Broader multifractal spectra recorded during the encounter with the deer dummy may indicate the heightened need for visual information in a critical situation. The second encounter was arguably less surprising due to the placement of the kid dummy on the side of the road on the last straight portion of the track, where it was clearly visible ahead (as demonstrated in Fig. 1). Very similarly to eye movements, recorded head movements also showed wider multifractal spectra during the events, indicating either a heightened need for visual information and/or a preparedness to counterbalance the forces of the quick maneuvers to avoid collision. One measure that clearly indexed the difference between human-driven and self-driving under normal circumstances was blinking frequency. Passengers blinked with significantly lower frequencies in the self-driving condition before encountering the dummies. During these novel experiences, the blinking rate in both human and self-driving conditions got even lower, diminishing the difference between modes of transportation. The pattern of the data suggests that under smooth driving conditions the novelty of self-driving induced a somewhat stronger inhibition of blinking. The unexpected events introduced an even higher effect in this direction, further lowering the blinking rate similarly in both driving modes.

The study presented here has certain limitations. We collected a large amount of data, but due to time and resource limitations, we could not include a wider range of analyses. For example, multifractal analyses of the EEG channels or fixation/saccade-based analyses of the eye tracking data remain to be conducted in follow-up reports. Another type of limitation is the way we reported aggregated and, therefore, simplified values of the events. While the analyses preserve the complexity of the movements, some aggregation is required to demonstrate the differences or changes over time as a function of events or conditions. One might also wonder whether the duration of the trials was long enough to measure reliable differences as the trials were relatively short. This again, was due to resource limitations, and running the experiment requires managing a large infrastructure, which is time-consuming. To a certain degree, we compensated the brevity of the experience with high sample rates that allowed for a fine-scale analysis of the events. On the other hand, it is important to note that some physiological responses do not require extended exposure or long timescales. Events that are of importance in vehicle navigation are generally fast. In this study we made an attempt to bring these two relatively quick structures to correspond. To conclude, our findings further establish real-world physiological measurements as a source of information in researching acceptance and usage of self-navigating technologies. The answer to our research question is that a combination of complex physiological measures can be applied to identify the differences in response to an unexpected event between self-driving and human driving. Our main results suggest that participants demonstrated a more withdrawal attitude in the self-driving condition, and that in critical road situations there is an enhanced need for visual information and preparedness. Incorporating physiological data into technological development is still in its infancy. Human-machine interaction^59^, stress and fatigue indicators^21,30–35,60^, cognitive load^47^, wearable medical monitoring^61^ are some of the more salient examples of creating feedback loops in development and utilization influenced by continuous measurement on user’s physiology. Widespread acceptance of new modes of transportation will likely benefit from these interactive loops especially in situations where developers need to monitor whether user expectations are being met.

## Data Availability

The datasets generated during and/or analyzed during the current study are available in the OSF repository, [https://osf.io/dhcaf/?view_only=a12e150a53a94585a32597c129ee5d4f]. Codes used in the analyses of the datasets are available from the corresponding author on reasonable request.
