# Author Correction: Passenger physiology in self-driving vehicles during unexpected events

**DOI:** 10.1038/s41598-025-97000-8

**Published:** 2025-04-10

**Authors:** Zsolt Palatinus, Miklós Lukovics, Márta Volosin, Zsolt Dudás, Szabolcs Prónay, Zoltán Majó-Petri, Henrietta Lengyel, Zsolt Szalay

**Affiliations:** 1 Department of Neuroscience and Cognition, Institute of Psychology, University of Szeged, Egyetem utca 2, Szeged, 6722 Hungary; 2 Research Centre for Natural Sciences, Institute of Cognitive Neuroscience and Psychology, Magyar Tudósok körútja 2, Budapest, 1117 Hungary; 3 Department of Economic Development, Faculty of Economics and Business Administration, University of Szeged, Szeged, Hungary; 4 Department of Business Studies, Faculty of Economics and Business Administration, University of Szeged, Szeged, Hungary; 5 Department of Automotive Technologies, Budapest University of Technology and Economics, Budapest, Hungary

Correction to: *Scientific Reports* 10.1038/s41598-024-81960-4, published online 06 March 2025

The Funding section in the original version of this Article was omitted. The Funding section now reads:

This research was funded by National Research, Development and Innovation Office – NKFIH, OTKA K137571.

The original Article has been corrected.

